# Effects of ascorbic acid on α-l-arabinofuranosidase and α-l-arabinopyranosidase activities from *Bifidobacterium longum* RD47 and its application to whole cell bioconversion of ginsenoside

**DOI:** 10.1007/s13765-015-0113-z

**Published:** 2015-08-27

**Authors:** Seockmo Ku, Hyun Ju You, Myeong Soo Park, Geun Eog Ji

**Affiliations:** Department of Food and Nutrition, Research Institute of Human Ecology, Seoul National University, 222 Dong 524Ho, Seoul, 151-742 Republic of Korea; Department of Hotel Culinary Arts, Yeonsung University, Anyang, 430-749 Republic of Korea; Research Center, BIFIDO Co. Ltd., Hongcheon, 250-804 Republic of Korea; Laboratory of Renewable Resources Engineering, Department of Agricultural and Biological Engineering, Purdue University, West Lafayette, IN 47907-2022 USA

**Keywords:** α-l-Arabinofuranosidase, α-l-Arabinopyranosidase, Ascorbic acid, *Bifidobacterium*, Bioconversion, Ginsenosides

## Abstract

*Bifidobacterium longum* RD47 was cultured in 24 kinds of modified MRS broths containing various ingredients to select the most promising source that induces microbial enzymes. Among the various ingredients, ascorbic acid significantly enhanced α-l-arabinofuranosidase and α-l-arabinopyranosidase activities in *Bifidobacterium longum* RD47. Addition of 2 % ascorbic acid (w/v) to MRS showed the maximum enzyme activities. Both whole cell and disrupted cell homogenates showed efficient ρ-nitrophenyl-β-d-glucopyranoside and ρ-nitrophenyl-β-d-glucofuranoside hydrolysis activities. The initially enhanced α-l-arabinopyranosidase and α-l-arabinofuranosidase activities by ascorbic acid were maintained over the cell disruption process. The optimal pH of α-l-arabinofuranosidase and α-l-arabinopyranosidase was 5.0 and 7.0, respectively. Both enzymes showed the maximum activities at 40.0 °C. Under the controlled condition using *Bifidobacterium longum* RD47, ginsenoside Rb2, and Rc were converted to ginsenoside Rd.

## Introduction

Panax ginseng (*Panax ginseng* C. A. Meyer) has been widely regarded as an important oriental plant medicine in East Asia since cultivation started around 11 bc (Jia and Zhao 2009). Ginseng contains various phytochemicals such as polyacetylenes, polyphenolic compounds, and ginsenosides (saponins). Among these phytochemicals, ginsenosides generally exhibit the pharmacological and nutraceutical effects of ginseng (Nagai et al. 1972; Karikura et al. 1991).

Previous research has identified approximately 40 types of ginsenosides and their quantities in ginseng (Kim et al. 1987). Ginsenosides can be divided into two main categories based on their aglycone: the protopanaxadiol groups (Ra, Rb1, Rb2, Rb3, Rc, Rd, Rg3, F2) and the protopanaxatriol groups (Re, R1, R2, Rf, Rg1, Rg2, F1, Rh1). These two groups of ginsenosides exhibit different pharmacological and nutraceutical effects on human health (Sakakibara et al. 1975; Shibata et al. 1976; Odashima et al. 1985; Toda et al. 1990; Scaglione et al. 1990; Wu et al. 1992). Among the various ginsenosides, ginsenoside Rd (Rd), the deglycosylated form of Rc and Rb2, has been regarded as a key indicator for identifying the quality of ginseng (Ye et al. 2013; Zhou et al. 2014). Several studies recently reported the wound-healing, neuroprotective, and anti-inflammatory uses of Rd itself (Ye et al.^ 2009^; Wang et al. 2012; Zhang et al.^ 2012^; Kim et al. 2013).

As reported by Tawab et al. (2003), the conversion of ginsenosides into deglycosylated form is crucial for its in vivo biological activity. Various methods (e.g., chemical treatment, mild acid hydrolysis, and alkaline cleavage) have been developed as ways to convert ginsenosides into deglycosylated form (Han et al. 1982; Bae et al. 2003; Ko et al. 2003). However, these methods produce significant amount of by-products (e.g., epimerization, hydration, and hydroxylation) (Chen et al. 1987; Elyakov et al. 1993; Chi and Ji 2005; Chi et al. 2005). In order to resolve these problems, numerous studies in both academia and industry use probiotic enzymes to transform ginsenosides into aglycones (e.g., Hasegawa et al. 1997; Bae et al. 2000; Ko et al. 2000; Ko et al. 2003; Bae et al. 2004). For example, several Korean food and pharmaceutical conglomerates have applied for patents to achieve ginseng market dominance over the last several years (Table 1). Nowadays, the size of the Korean ginseng market is estimated to be $1.14 billion (Baeg and So 2013).

**Table 1:** Intellectual properties of Korean foods and drug companies related to bioconversion of ginsenosides

Company	Patent title	Right registration
Nongshim Co., Ltd.	Saponin-biotransforming activity and processes for preparing fermented ginseng using the same	4 July 2013
	Korean intestine-derived microorganisms having saponin-biotransforming activity and processes for preparing fermented ginseng using the same	26 November 2010
Lotte Chilsung	Fermented ginseng containing bio-conversed ginsenoside metabolites increased by co-fermentation of fungi and lactic acid bacteria	6 March 2014
Chong Kun Dang Pharmaceutical Corp.	Korean intestine-derived microorganisms having saponin-biotransforming activity and processes for preparing fermented ginseng using the same	26 November 2010
Woongjin Foods Co., Ltd.	Novel microorganism for red ginseng fermenting, ferment solution and fermentative red ginseng drink using the same	5 August 2013
Korea Yakult Co., Ltd.	A method of preparation for fermented red ginseng using conversion by enzyme mixture and fermentation by lactic acid bacterium and the products containing fermented red ginseng manufactured thereof as effective factor	13 June 2014
Daesang Corp.	A novel strain of kimchi lactic acid bacteria having ginsenoside Rg3 enrichment activity and methods for preparing fermented ginseng using the strain	5 June 2014

In order to convert ginsenoside Rc and/or Rb2 into Rd, α-L-arabinofuranosidase (Abf) and α-L-arabinopyranosidase (Abp) have been cloned in *Escherichia coli* (Lee et al.  2011; An et al. 2012). However, from a marketing and food safety point of view, using genetically modified organism and *E. coli* has practical limitations for use in the food industry. Several studies have shown that the addition of specific nutrients can considerably change microbial enzyme activities; in this case, the induced enzyme can be applied to ginsenoside conversion (Crociani et al. 1994; Degnan and Macfarlane 1995; Salyers et al. 1977; Tzortzis et al.^ 2003^; Hsu et al. 2005; Ku et al. 2011).

Herein, we aim to show the optimal condition (i.e., the concentration of ascorbic acid and ginseng extract, temperature, cell disruption step, and pH) to improve Abf and Abp activities in *Bifidobacterium longum* RD47 (BL47). The induced Abf and Abp were applied to convert ginsenoside Rb2 and Rc into Rd.

## Materials and methods

### Materials

Panax ginseng roots were purchased from a local grocery store in Korea. Chiro-inositol and pinitol were provided by Amiocogen Co., Ltd. (Korea). Acetonotrile, methanol, and water were purchased from J. T. Baker^®^ (USA). Ginsenoside standard Rb2, Rc, and Rd were purchased from BTGin Co., Ltd. (Korea). Yeast extract, proteose peptone, beef extract and deMan, Rogosa, Sharp (MRS) media were purchased from Becton, Dickinson and Company (BD) (USA). Glucose-free MRS was formulated according to the manual of microbiological culture media (Difco^TM^ and BBL^TM^ Manual 2009; Ku et al. 2009, 2011). The glucose-free MRS contained 10 g proteose peptone, 10 g beef extract, 5 g yeast extract, 1 g polysorbate 80, 2 g ammonium citrate, 5 g sodium acetate, 0.1 g magnesium sulfate, 0.05 g manganese sulfate, and 2 g dipotassium phosphate in 1 l of distilled water. The pH of the broth was 6.5 ± 0.2 at 25 °C and 2 % of agar was added if needed.

### Cell growth condition

In order to select a promising nutrient for the enzyme induction, various modified MRS broths containing different carbon sources were designed (Table 2). The pH of all broths was adjusted to 7.0 via the addition of sodium hydroxide, and all the broths were sterilized using 0.2 µm syringe Ersatz-Membranfilter (BRAND^®^, Germany). After two successive transfers in the MRS broth, 1 % (v/v) of activated BL 47 was inoculated into each modified MRS broth and grown anaerobically at 37 °C for 18 h. The viable cell counts were determined by plating on MRS containing 2 % agar (BD, USA) under anaerobic conditions. Cell growth rates were measured optically using a spectrophotometer at 600 nm (Model Benchmark, Bio-Rad, Japan) (Table 2).

**Table 2:** Modified MRS broths tested in the experiment

No.	Ingredient	*X*/*X*_M_	No.	Ingredient	*X*/*X*_M_	No.	Ingredient	*X*/*X*_M_	No.	Ingredient	*X*/*X*_M_
Glucose-free MRS + 2 % (w/v) of nutrient											
1	l-Arabinose	0.74 ± 0.05	2	Lactose	0.59 ± 0.04	3	Raffinose	0.77 ± 0.04	4	Fructose	1.35 ± 0.06
5	Glucose	0.95 ± 0.06	6	Maltose	1.13 ± 0.03	7	Sucrose	1.54 ± 0.07	8	Xylose	0.64 ± 0.02
MRS + 2 % (w/v) of nutrient											
9	l-Rhamnose	0.91 ± 0.01	10	Raffinose	0.95 ± 0.02	11	Chiro-inositol	0.95 ± 0.01	12	Pinitol	0.89 ± 0.04
13	Fructose	1.01 ± 0.01	14	Maltose	0.92 ± 0.01	15	Sucrose	1.13 ± 0.04	16	Xylose	0.57 ± 0.04
17	Cellobiose	0.93 ± 0.02	18	Ribose	0.83 ± 0.01	19	Sucralose	0.9 ± 0.04	20	Mannose	0.87 ± 0.06
21	Lactic acid	0.61 ± 0.01	22	Glycine	0.88 ± 0.02	23	Asc-acid	1.02 ± 0.03	24	Cit-acid	0.52 ± 0.02

*X*/*X*_M_ relative microbial growth rates in which *X*_M_ growth rate at commercial MRS, *X* growth rate at modified MRS, *Asc*-*acid* ascorbic acid, *Cit*-*acid* citric acid

### Enzyme assay

Enzyme activities were measured for three different samples: whole, lysed, and disrupted cells. Whole cell suspension was prepared as previously described (Park et al. 2012). Cell lysis step was carried out to extract microbial enzyme from the whole cell using lysis solution as described in Ku et al. (2011). Disrupted cell suspension was prepared by the cell sonicator set at 45 amplifications for 3 min at 4 °C. For the enzyme reaction, 5 mM of ρ-nitrophenyl-β-D-glucopyranoside (pNPP), and ρ-nitrophenyl-β-D-glucofuranoside (pNPF) (Sigma, St. Louis, Mo., U.S.A.) were used. The released pNP was measured at 405 nm (Model Benchmark, Bio-Rad, Japan) after enzyme reaction at 37 °C. Enzyme activity was evaluated using the following equation:

$${{\ln \left[ A \right]} \mathord{\left/ {\vphantom {{\ln \left[ A \right]} {\left[ A \right]_{\text{M}} = - kt}}} \right. \kern-0pt} {\left[ A \right]_{\text{M}} = - kt}}$$

in which *A* = enzyme activity of BL47 cultured at modified MRS at time t; *A*_M_ = enzyme activity of BL47 cultured at normal MRS; *k* = rate constant; and *t* = time (min) (Ximenes et al. 2011).

### Determination of the optimal enzyme condition (ascorbic acid concentration, ginseng extracts concentration, pH, cell disruption time, and temperature)

To determine the optimal concentration of ascorbic acid, BL47 was anaerobically grown in MRS with 0–5 % (w/v) of ascorbic acid at 37 °C for 18 h. After determination of the optimal ascorbic acid concentration, 0–55 % (v/v) of ginseng extracts were added to the modified MRS broth containing 2 % ascorbic acid (w/v). The activated BL47 was inoculated to each media and anaerobically grown at 37 °C. One ml (5 × 10^8^ CFU/ml) of the cell suspension was harvested, washed twice in PBS, and then re-suspended in 4 ml of PBS at 37 °C. During the cell sonication process, enzyme activity was evaluated at 30 s interval as described above. The degree of cell disruption was measured by the optical density at 600 nm. The optimal pH and temperature of Abf and Abp were determined by the aforementioned method (Ku et al. 2011).

### Treatment of ginseng extracts using disrupted cell suspension

Ginsenosides were extracted from the ginseng root using the method described in our previous study (Kim et al. 2008). The disrupted cell suspensions from 5 × 10^8^ CFU/ml were mixed with the ginseng extracts at the ratio of 19:1 (v/v) and incubated at 37 °C. The cell-ginseng extract suspensions were collected after 3, 6, 9, and 12 days and evaluate the bioconversion of ginsenosides through TLC analysis (Park et al.  2012).

### Addition of ginseng extracts to MRS broth supplemented with ascorbic acid and ginsenosides conversion

Five to 55 % (v/v) of ginseng extracts were added to MRS + 2 % ascorbic acid (w/v) broth. The initial pH of all broths was adjusted to 7.0 by adding sodium hydroxide. Activated BL 47 was anaerobically cultured at 37 °C for 7 days without shaking. The whole cell and ginseng extract suspensions were collected after 2, 3, 4, 5, 6, and 7 days to evaluate the bioconversion of ginsenosides using the TLC analysis (Park et al. 2012). The changed Abf and Abp activities by the concentration of ginseng extracts were determined using our previous method (Ku et al. 2011).

### Statistical analysis

For the statistical evaluation of cell growth rates and changed enzyme activities, the analysis of variance (ANOVA) was applied using the program Minitab^®^ 16, and the Tukey’s test was applied for the post hoc comparison. Significant differences were considered at *p* < 0.05.

## Results and discussion

### Induction of Abf and Abp using ascorbic acid

**Fig. 1:**
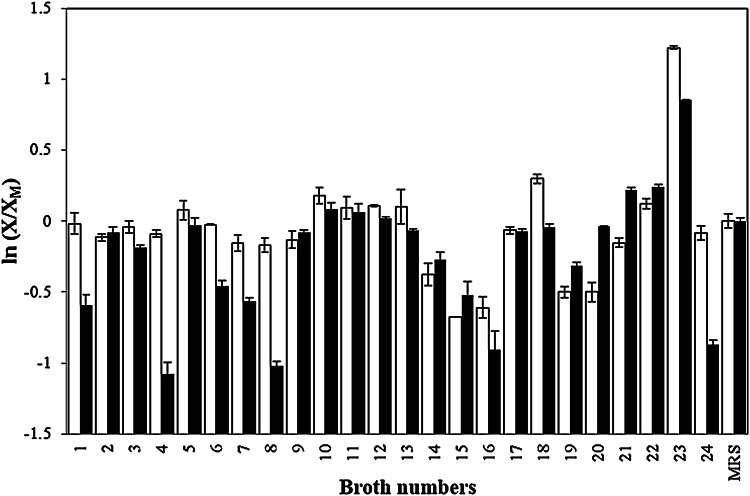
Relative enzyme activities of *Bifidobacterium longum* Rd47 cultured in the various broths (*n* = 3). Cell lysis solution was treated to samples. *Error bars* standard deviation. *White bars* α-arabinofuranosidase, *black bars* α-arabinopyranosidase

In our previous work, various carbon, nitrogen, and ion sources were added to the microbial culture broth and determined the optimal α- and β-galactosidases production from BL47 (Han et al. 2014). In this study, we aimed to investigate which sources were effective inducers for the Abf and Abp and whether any of the sources could promote the growth of BL47 (Table 2). As sole carbon sources, L-arabinose, lactose, and xylose (#1, 2 and 8) were ineffective for the growth of BL47. Fructose and maltose showed a slight enhancement (#4 and 6). BL47 showed the best growth when sucrose (#7) was added. There were no statistically significant growth differences between commercial MRS (control) and modified MRS containing glucose (#5) (*p* < 0.05), which demonstrates that commercial MRS and lab-made MRS have a similar effect on cell growth (Fig. 1).

**Fig. 2:**
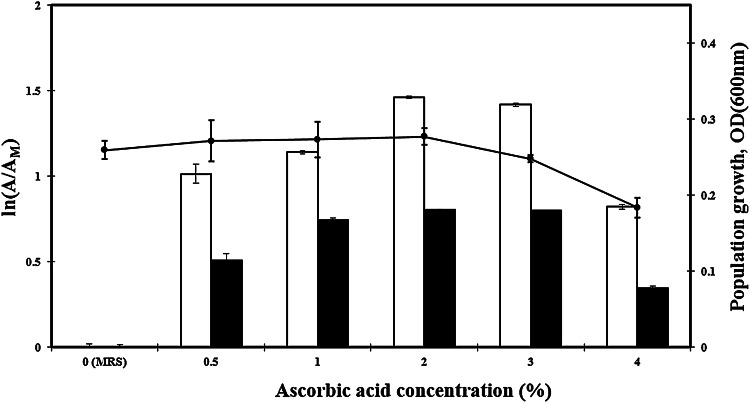
The effect of ascorbic acid on the production of α-L-arabinofuranosidase and α-L-arabinopyranosidase from *Bifidobacterium longum* Rd47 (*n* = 3). Cell lysis solution was treated to samples. *Error bars* represent standard deviation. *White bars* α-arabinofuranosidase, *black bars* α-arabinopyranosidase, *black circles* population growth

Several studies have shown that the addition of certain organic acids to culture media can improve the cell viability by neutralizing hydrogen peroxide and reducing redox potential (Brewer et al. 1977; Collins and Hall 1984; Ku et al.  2011). Interestingly, the addition of some organic acids to culture media modified the morphology of *Bifidobacterium* spp. by ion chelation during fermentation (Kojima et al. 1968, 1970; Ku et al. 2009). In this work, the addition of 2 % (w/v) lactic acid (#21) and citric acid (#24) to commercial MRS showed a decrease in cell viability (*p* < 0.05). However, the degrees of the BL 47 growth were not significantly affected by the presence of ascorbic acid (*p* > 0.05), as compared to those cultured in normal MRS (Fig. 1). The microscopic morphology of BL47 was not changed by ascorbic acid (data not shown). Both Abf and Abp activities of BL47 were outstandingly increased by adding 2 % of ascorbic acid (w/v) (*p* < 0.05). For further examination of the role of ascorbic acid, 0–4 % (w/v) of ascorbic acid was added to commercial MRS (Fig. 2). As a result, the degree of enzyme activity increased as the concentration of ascorbic acid increased up to 2 %. Conversely, BL 47 cultured in media containing 4 % of ascorbic acid showed significantly decreased enzyme activity (*p* < 0.05) and decreased growth (*p* < 0.05).

These results suggested that ascorbic acid can enhance Abf and Abp activity and were optimal at 2 %. Because majority of probiotic bacteria are anaerobic microorganisms, it is common in the food industry to add ascorbic acid into commercial probiotic products in order to scavenge oxygen. Several studies have demonstrated the effects of ascorbic acid on lactic acid bacteria (Dave and Shah 1997; Talwalkar and Kailasapathy  2004; Santiesteban-López et al. 2013; Shu et al. 2013). These studies focused on evaluating changed cell growth rates to determine best conditions for the yield of lactic acid bacteria. The concentrations of ascorbic acid used in their experiments were relatively lower (<0.1 % w/v) than in our experiment (2 % w/v). The present study reports a newly observed enhancement of Abf and Abp activities from the genus *Bifidobacterium* by ascorbic acid.

### Optimal pH, temperature, and disruption conditions for Abf and Abp

Some microbial enzymes produced from lactic acid bacteria showed high enzyme activity in the acidic conditions (Ku et al. 2011). The optimal pHs of Abp in *B. breve* K-110 and *B. longum* H-1 were 5.8 and 6.8, respectively (Shin et al. 2003; Lee et al. 2011). The optimal pHs of Abf activity in *B. breve* K-110 and *B. longum* H-1 were 4.5 and 4.7, respectively. The maximum Abf and Abp activities of the BL47 cultured in MRS were observed at pH 6.0 and 5.0, respectively, whereas those cultured in MRS broth containing 2 % of ascorbic acid (w/v) were observed at pH 7.0 and 5.0 (*p* < 0.05), respectively.

**Fig. 3:**
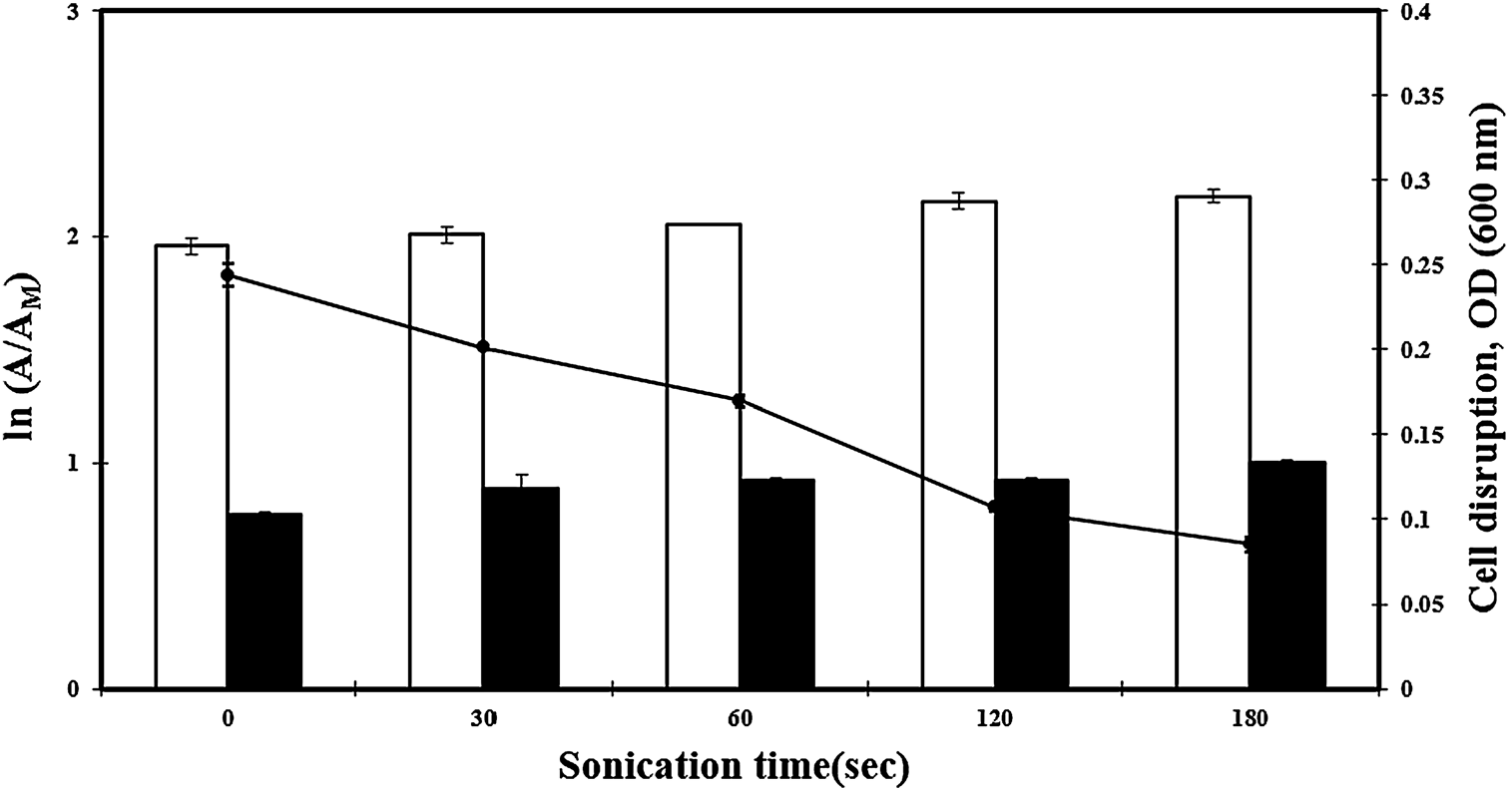
The effect of cell disruption (Sonication) on α-l-arabinofuranosidase and α-l-arabinopyranosidase activities from *Bifidobacterium longum* Rd47 (*n* = 3). *Error bars* represent standard deviation. *White bars* α-arabinofuranosidase, *black bars* α-arabinopyranosidase, *black circles* degree of cell disruption

The maximum Abf and Abp activities were observed at 40 °C for BL47 within our experimental range of 4–70 °C and about 60 % of both Abf and Abp activities were detected after incubation at 60 °C for 10 min compared to the maximum activity (data not shown). During the cell sonication process, the degree of cell disruption was increased while the optical density of cell suspension was gradually decreased at 600 nm (Fig. 3). There was no statistically significant loss of the Abf and Abp activities (*p* > 0.05) during this process. The whole cell suspension efficiently hydrolyzed ρ-nitrophenyl-β-d-glucopyranoside and ρ-nitrophenyl-β-d-glucofuranoside. These results suggest that both enzymes apparently have high resistance to physical disruption.

Cell disruption process is essential to use cytosolic enzymes for the bioconversion (Chi and Ji 2005; Chi et al. 2005; Yan et al. 2008; Noh and Oh 2009; Yoo et al. 2011). However, these processes are time consuming and labor intensive. In our previous work (Ku et al.^ 2011^; Park et al. 2012), we reported successful hydrolysis of glycosides using whole cell without cell disruption process. This minimal microbial process may lead to cost reduction, an important practical application in the food industry.

### Bioconversion of ginsenosides

**Fig. 4:**
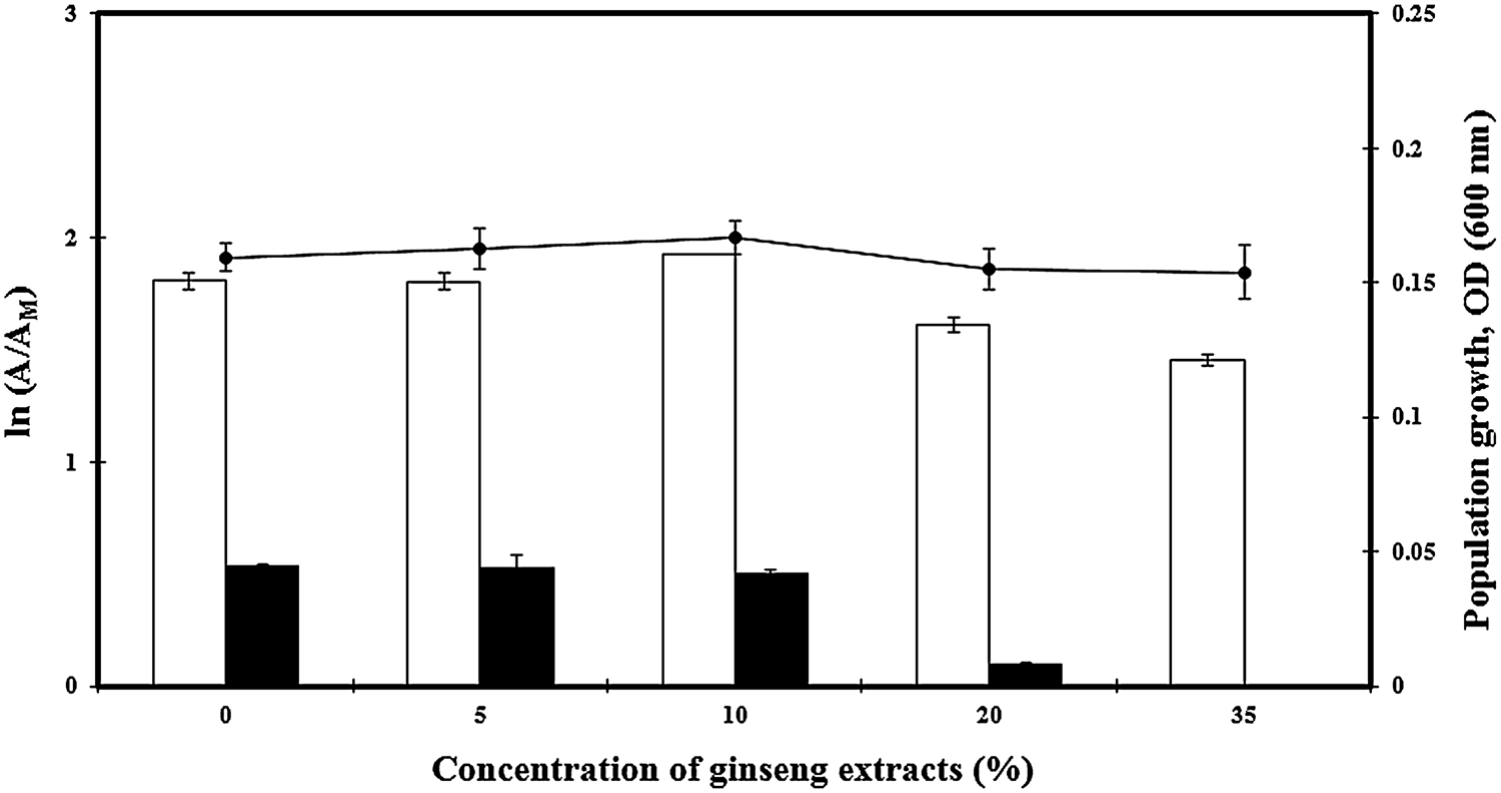
The effect of ginseng extracts on the production of α-l-arabinofuranosidase and α-l-arabinopyranosidase from *Bifidobacterium longum* Rd47 (*n* = 3). Enzyme activities were evaluated without disruption step. *Error bars* standard deviation. *White bars* α-arabinofuranosidase, *black bars* α-arabinopyranosidase, *black circles* population growth

The use of microbial crude enzymes can reduce food processing cost, as compared to the use of purified enzymes (Singh et al. 2013). Based on the presently determined optimal conditions [ascorbic acid concentration: 2 % (w/v); temperature: 40 °C; pH: 5], the disrupted BL 47 homogenates (5 × 10^8^ CFU/ml) and the whole cell suspension of BL 47 (5 × 10^8^ CFU/ml) were applied to the hydrolysis of natural substrates. Ginsenosides Rb2 and Rc are differentiated from Rd by the presence of α-l-glucopyranoside and α-l-glucofuranoside, respectively; therefore, aglycone of ginsenosides Rb2 and Rc is the same as that of ginsenoside Rd (Shin et al. 2003). The whole cell suspension converted both Rb2 and Rc into Rd; however, the disrupted cell extracts only converted Rb2 to Rd. The effect of BL47 enzymes on the experimental ginsenosides was slightly different from its effects on pNP substrates. This difference may be caused by the direct application of crude enzyme homogenates (i.e., the whole cell and disrupted cell suspensions) into the bioconversion step without protein purification. Similar results were reported with microorganisms *Flavobacterium johnsoniae* and *Cladosporium cladosporioides* (Hong et al. 2012; Wu et al. 2012). When BL47 was cultured in modified MRS containing 2 % ascorbic acid and various levels of ginseng extracts, both Abf and Abp showed maximum activities when the ginseng extracts were 10 % (v/v) without significant changes in growth rate (*p* > 0.05) (Fig. 4). However, the enzyme activities were gradually decreased by adding additional ginseng extract to the broths.

**Fig. 5:**
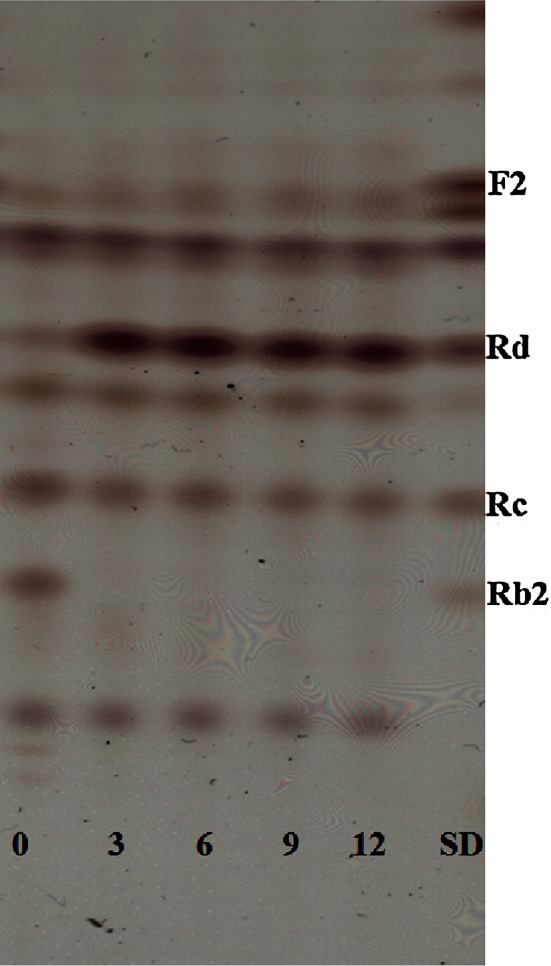
TLC profile of ginsenosides conversion using disrupted cell

**Fig. 6:**
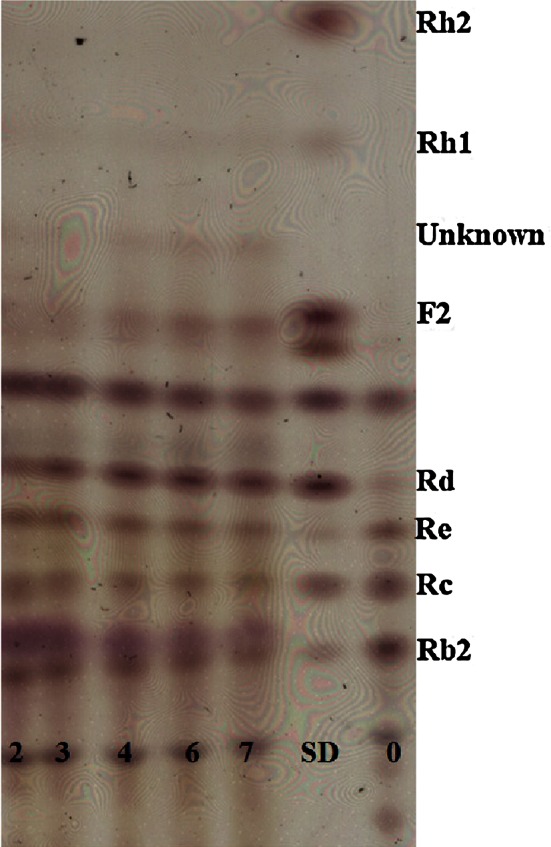
TLC profile of ginsenosides conversion using whole cell

During the processing step to make ginseng extracts, a high level of carbohydrates can be extracted from ginseng because more than 60 % of the ginseng root consists of carbohydrates (Van et al. 2009; Choi et al. 2014). Several studies report that the addition of a high concentration of carbon sources to culture media decreased the induced enzyme activity (van der Veen et al. 1994; Gielkens et al. 1999; Gueimonde et al. 2007; Hetta et al. 2014). Sánchez and Hardisson (1980) hypothesized that this enzyme inhibition may be the result of catabolite repression and inactivation, or the reduced usability of the inducer. The TLC profile of transformed ginsenosides by using the whole cell suspension of BL47 showed a transformation of ginsenoside Rc and Re to Rd. We also detected ginsenoside F2 and unknown faint bands (Figs. 5, 6).

This study revealed that the addition of 2 % of ascorbic acid to MRS media caused a significant increase in Abf and Abp activities of BL47. We also showed the optimal conditions for the induced enzymes. Based on our results, we applied the whole cell and disrupted cell homogenates to the bioconversion of ginsenosides in order to use this process in industrial applications. The bioconversion using whole and living cell in media containing ginseng extracts is not perfectly completed; however, the potential for reducing production cost has been approved. Our protocol is more practical for the bioconversion of ginsenosides than conventional methods, which usually include numerous procedures (such as enzyme purification, cell disruption, gene work). Further work in the molecular level should be conducted in order to investigate the effect of ascorbic acid on BL 47.
